# Bibliography

**Published:** 1903-02

**Authors:** 


### Bibliography.

How to succeed in the Practice of Medicine. By Joseph McDowell Mathews, M.D., LL.D., President of American Medical Association, 1898-99. Author of "Mathews on Diseases of the Rectum;" Ex-President Mississippi Valley Medical Association; Professor of Surgery, Hospital Medical College, etc. John P. Morton & Co., Louisville, 1902.

It is not often a reviewer of a medical or dental book reads it with real pleasure. He probably regards it as a duty that cannot be avoided if he will do justice to the writer, publisher, and himself. In this instance, however, the writer found himself so infused with the spirit of the author and so invigorated with his delightful style and valuable ideas that he read the book through from first page to the last, and left it with the conviction that it should be in the hands of every young medical and dental graduate, and for the older practitioner it would possess intense interest; for between the lines he could read his early experiences, recalling amusing recollections, and at others pointing to mistakes and neglected opportunities.

The book has been written for medical graduates, but it applies throughout with equal force to all professional men, and the young dental graduate can read it with interest and instruction.

The author says, in his preface, "The book is enlarged that it may interest all members of the medical profession, and the laity are earnestly requested to read it. . . . It has been painful to me to see many of my professional friends die and leave their families in actual want. The reason is plain that it was either their own fault or the fault of their clientele, and how to prevent this happening to others is the main object of the book."

The author is an ex-president of the American Medical Association and a medical author of national reputation, and anything he may write upon the broad subject of "How to succeed in the Practice of Medicine" should find attentive readers everywhere.

He utters these great truths when he writes, "After an experience extending over twenty-five years as teacher in a medical college, I beg to submit the following conclusions:

"1. The better educated one is, the better prepared he is to understand the teachings of the great science of medicine.

"2. Upon the foundation of learning as laid by a common-school education men have attained to the highest positions in the medical profession, both as teachers and practitioners.

"3. Many men with the highest education that could be afforded by Harvard, Yale, or Princeton have signally failed as practitioners of medicine.

"4. Many men of self-education have attained to the highest positions known in the medical profession."

These conclusions are equally applicable to dentistry, and are in entire accord with the writer's forty years' experience as a teacher in his profession. The result is all dependent upon the man, but, given equal ambitions and natural tastes, the man with the superior foundational education will outstrip the one lacking it, for the latter is always struggling to overcome the defects of his early education.

To the "young doctor who changes his location several times before becoming permanently settled" he has this to say:

"We are to presume that you visit the place recommended; it has been told you that it is a first-class location because there are no doctors there; no, not within miles of it. . . . It is an insult to your intelligence to hint such a reason. Are you afraid of honest competition? If so, you had better go back to school. . . . Do you not want the companionship and the help in time of need of some good, kind brother in the profession?"

This will apply equally as well to the young dentist. It is not ordinarily the most favorable location where there is not a dentist for miles. It is very certain to be a place where money is limited and banks are unknown, and without these prerequisites to success the work of the dentist will be regarded as a luxury not attainable by the impecunious population. Taken as a whole, it is probable that the most successful dentists are those in towns of from forty to fifty thousand population.

It is not possible to quote all the good points in this book. It is rich in anecdote, and the illustrations, although few in number, must appeal to every practitioner, especially that in which the young graduate is hanging out the first sign. Has any one ever forgotten this first sign? How formidable it looked in broad daylight, and how fearfully and anxiously the first patient is looked for as the result of this wonderful announcement!

Every young medical and dental graduate could read and study this book with profit, and careful attention to its instructions may be the means of leading many to pleasant surroundings, delightful homes, and eventually into a remunerative practice.

The publishers' work is everything to be desired, in type, paper, and general make-up.

A Manual of the Injuries and Surgical Diseases of the Face, Mouth, and Jaws. By John Sayre Marshall, M.D., Former Professor of Dental Pathology and Oral Surgery, and Emeritus Professor of Oral Surgery of the Dental Department of Northwestern University; Attending Oral Surgeon to St. Luke's Hospital, Mercy Hospital, and Baptist Hospital, of Chicago; President of the Examining Board for Dental Surgeons, United States Army. Second edition, revised and enlarged. The S. S. White Dental Manufacturing Company, Philadelphia, 1902.

This book was so thoroughly reviewed in its first edition in this journal that the favorable opinion then held can only be transferred to the second, with the opinion that it has been greatly improved by some very valuable additions and with much, not so valuable in the first edition, eliminated. The questions following each chapter have all been removed. These were of no real value, and had the effect of lowering the otherwise high standard of the book. Another removal that especially appeals to the reviewer is the elimination of authorities quoted in almost every paragraph in the first edition. The constant reiteration of the same names became very monotonous.

The author, Dr. Marshall, is thoroughly at home when writing of surgical diseases and surgery, and the reviewer can only repeat what was said in the first edition with more emphasis in the second, that this book is not likely to have any superior, upon the subjects on which it treats, in this generation. It meets all demands both in its pathology and in its operative procedures.

The several chapters have been greatly improved by valuable illustrations. This is especially true of that on "Necrosis," which was rather weak in the first edition, but sufficiently extended in this.

Chapter XXXI., on "Leucoplakia," is altogether new. This pathological condition was very briefly alluded to in the first edition, but in this it is one of the most interesting of the book. The same can be said of "Actinomycosis Hominis," Chapter XXXV. It is fully illustrated, and explains very satisfactorily this peculiar phase of infection.

While the subject of "Tumors" is of great importance, it would seem that to give two hundred and seventeen pages to their consideration is an unnecessary extension of the size of the volume. It must be acknowledged, however, that the author has managed to keep the book down to the same number of pages as the first edition, a very important matter, as a bulky text-book is a serious evil.

The title-page remains with the word "Manual" at the head. This does not, in the reviewer's opinion, correspond with the high character of the book.

It is a satisfaction to find that this work has been appreciated, and that the demand has so early called for the second edition. That it will become a standard work upon the subjects treated there can be no question, thus demonstrating the oft-observed fact, that when an author attempts to write upon a subject, he must have lived through it in many years of experience, and this has certainly been true of the author of this valuable text-book.

A Text-Book of Surgical Principles and Surgical Diseases of the Face, Mouth, and Jaws, for Dental Students. By H. Horace Grant, A.M., M.D., Professor of Surgery and of Clinical Surgery in Hospital College of Medicine; Professor of Oral Surgery in the Louisville College of Dentistry, etc. Illustrated. W. B. Saunders & Co., Philadelphia and London, 1902.

The author says in his preface that it "is the object of this work to present to the student of dentistry a text-book that will succinctly explain the principles of dental surgery applicable to all operative procedures, and also to discuss such surgical lesions as are likely to require diagnosis and perhaps treatment by the dentist;" and further he states, "An exhaustive analysis of the phenomena of inflammation and repair, as well as of the pathology of the essential processes of acute surgical diseases, is not desired by the student of dentistry during his college days." Perhaps if the student were consulted he would agree to this proposition, but where, may it be asked, will he acquire this important knowledge if not in the college? This seems to be a most unfortunate position for an author to take at the very opening preface of his book, but it is the old story, that a dentist requires but little outside of practical procedures, and some teachers, acting upon this, proceed to ladle out this knowledge in the weakest possible portions. This is certainly not the position the teacher or author should hold towards the undergraduate.

The author begins his work with a chapter on "Bacteriology and Surgical Principles." Seven pages are given to the first subject, but the reviewer fails to find any space occupied with the latter, surgical principles. This is not very important, but indicates carelessness in the preparation of the book.

Chapter II. is devoted to "Inflammation," and the author has certainly carried out his idea promulgated in the preface that the dental student does not need "an exhaustive analyses" of a subject, for if the said student is earnest in his efforts to acquire a knowledge of inflammation, he will be forced to examine books of wider range than this. The histological phenomena following hyperæmia are not explained satisfactorily; indeed, it is safe to assume that it would be impossible for a student to secure a very clear idea of what inflammation means through the description given by the author.

While this is a marked defect in the chapter on this foundation subject, there is much to commend in the author's brief descriptions, both here and throughout the book. To one conversant with the subjects treated they appeal, as condensed statements, but the book is not written for these, but for students. The mind at this stage needs, above all things, detail. He must know the entire subject, or an attempt at instruction had better be omitted.

On page 29 the author recommends: "When hidden suppuration is suspected . . . the use of warm poultices" is recommended. He probably had not in view an alveolar abscess when this was written, but it would be rather dangerous advice to give a dental class without some further explanation.

The reviewer, in reading this volume of much valuable matter and, upon the whole, well prepared, has been constantly impressed with the feeling that condensation has been carried to almost unpardonable limits. For instance, sterilization of instruments is condensed into six lines, and the dental student is informed that

"dental instruments that are being used upon a septic case may be rapidly sterilized by dipping them in wood alcohol and passing them through a flame."

In the chapter on "wounds, including Shock," this, while reasonably well described, does not include the recognized danger of shock following dental operations. The author would do well to read Black upon this subject in the "American System of Dentistry.

The chapter on "Hemorrhage" is well treated, but the author seems to rely very much on Monsel's solution of iron to check hemorrhage. Very few dentists would think of making use of this agent in serious cases, for its well-known effect of lowering the tone of the vessels and inviting secondary hemorrhage makes its use very objectionable.

The general subjects of the book are generally well treated, but it is hoped when a second edition is called for that the promise of the preface that the "principles of dental surgery applicable to all operative procedures" will be more fully carried out. The author will remember that dental students in the twentieth century demand and should receive the same treatment that is accorded the medical undergraduate. Anything less means partial culture, and partial culture is only one remove from absolute ignorance.

The reviewer has felt called to. rather sharply criticise the general method of this book, in the hope that a really good book may be made of it in the second edition. The author has the right idea of what is generally needed, but fails through a narrow conception of what dentistry requires in the teaching of the present time.

